# Metformin for Clozapine Associated Obesity: A Systematic Review and Meta-Analysis

**DOI:** 10.1371/journal.pone.0156208

**Published:** 2016-06-15

**Authors:** Dan J. Siskind, Janni Leung, Anthony W. Russell, Daniel Wysoczanski, Steve Kisely

**Affiliations:** 1 School of Medicine, The University of Queensland, Brisbane, Qld, Australia; 2 Metro South Addiction and Mental Health Service, Brisbane, Qld, Australia; 3 QCMHR, School of Public Health, The University of Queensland, Brisbane, Qld, Australia; 4 Institute for Health Metrics and Evaluation, University of Washington, Seattle, United States of America; 5 Department of Endocrinology, Princess Alexandra Hospital, Brisbane, Qld, Australia; 6 Griffith Institute of Health, Griffith University, Brisbane, Qld, Australia; 7 Departments of Psychiatry, Community Health and Epidemiology, Dalhousie University, Halifax, Canada; University of Lancaster, UNITED KINGDOM

## Abstract

**Background:**

Although clozapine is the gold-standard for treatment refractory schizophrenia, it has the worst metabolic profile of all antipsychotics. This is partly mediated by clozapine’s impact on glucagon-like peptide (GLP-1). There is an absence of robust evidence for effective treatments for clozapine associated weight gain and metabolic syndrome. Metformin, with its role in increasing GLP-1 may aid weight loss among people on clozapine.

**Methods:**

We conducted a systematic-review and meta-analysis of metformin versus placebo for change in weight and metabolic syndrome for people on clozapine without diabetes mellitus. We searched the Cochrane Schizophrenia Group’s trial register, Pubmed and Embase, as well as the following Chinese databases: the Chinese Biomedical Literature Service System and China Knowledge Resource Integrated Database. This was supplemented by hand searches of key papers.

**Results:**

Eight studies, of which three were from Chinese databases, with 478 participants were included. We found that metformin was superior to placebo in terms of weight loss (-3.12kg, 95%CI -4.88kg to -1.37kg) and BMI (-1.18kg/m^2^, 95%CI -1.76kg/m^2^ to -0.61kg/m^2^). Metformin significantly improved three of the five components of metabolic syndrome; waist circumference, fasting glucose and triglycerides. Sensitivity analysis on study quality and duration did not greatly impact results.

**Conclusions:**

Metformin led to clinically meaningful weight loss among people on clozapine, and may reduce the rates of metabolic syndrome. Inclusion of metformin into the treatment protocols of people on clozapine, as tolerated, should be considered.

**Trial Registration:**

PROSPERO registration number: CRD42015029723

## Introduction

Life expectancy for people with schizophrenia is 16.4 years shorter than for the general population [1] with 35.1% of excess deaths attributable to cardiovascular disease and diabetes mellitus [1]. People with schizophrenia are at risk of developing cardio-metabolic disease for several reasons including a genetic predisposition to developing diabetes [2], reduced physical activity [3] and the use of antipsychotic medications [4].

Although antipsychotic medications are a core component of treatments for schizophrenia [5], approximately 20% of patients are treatment refractory, defined as non-response to adequate trials of at least two different antipsychotics [6]. In this situation, Clozapine is the most effective medication [7] although, unfortunately, it is associated with the highest rates of metabolic syndrome of all antipsychotics [4].

Metabolic syndrome is defined as having at least three of five abnormal results from the following parameters: waist circumference, fasting glucose, High Density Lipoprotein (HDL) cholesterol, Triglycerides, and blood pressure [8]. A recent meta-analysis reported that 51.9% of people on clozapine had metabolic syndrome compared to 28.2% for olanzapine and 27.9% for risperidone [4]. In a ten-year follow up of people commenced on clozapine in the USA 43% developed diabetes with a mean weight gain of over 13.5kg [9].

Although there is increasing evidence for the efficacy of physical activity interventions for people with schizophrenia [3], poor rates of uptake remains a barrier to their effectiveness. As a result, there has been growing interest in other interventions such as oral medication. A recent systematic review of treatments for weight gain associated with clozapine found limited evidence for the efficacy of, aripiprazole, fluvoxamine and topiramate [10]. At the time of their search, they identified two randomised control trials of metformin specifically for people on clozapine, with promising results, but these were insufficient to undertake a meta-analysis.

Among users of all anti-psychotics, there is increasing evidence for metformin for the management of weight gain [11]. Metformin is a biguanide antihyperglycaemic commonly used in the management of type 2 diabetes mellitus [12]. It causes mild weight loss in people without diabetes who are not on antipsychotic medications [13], and reduces fasting glucose, insulin and, triglyceride levels while increasing high-density lipoprotein (HDL) [14]. Its antihyperglycaemic properties are mostly attributed to suppression of hepatic gluconeogenesis and increased peripheral insulin sensitivity [12].

There is also evidence that metformin increases the production of Glucagon-like Peptide (GLP-1), an intestinal epithelium produced peptide, following food [15]. In turn, GLP-1 stimulates insulin secretion while inhibiting glucagon secretion, and also appears to regulate appetite by inducing satiety [16]. Metformin’s role in GLP-1 regulation is of particular relevance for people on clozapine as clozapine disrupts the glucagon-like peptide (GLP-1) pathway in the intestinal epithelium, thereby reducing GLP-1 levels [17]. As such, it is possible that metformin may have greater benefits for people on clozapine than for other anti-psychotics.

To date, no meta-analysis has specifically examined the impact of metformin on weight gain and metabolic syndrome associated with clozapine. We, therefore, undertook a systematic review and meta-analysis randomised control trials of metformin on weight and metabolic syndrome in people on clozapine.

In particular, our aim was to examine whether metformin was associated with changes in:

1. Body mass
2. metabolic syndrome subcomponents
  - Waist circumference (WC)
  - Fasting Blood Glucose (FBG)
  - HDL
  - Triglycerides (TG)
  - Blood Pressure (BP)

## Method

### Protocol and registration

This study was registered with PROSPERO (registration number: CRD42015029723), an international database of prospectively registered systematic reviews [18]. We followed the Preferred Reporting Items for Systematic Reviews and Meta-Analyses (PRISMA) statement recommendations for the background, search strategy, methods, results, discussion and conclusions [19]. Ethical approval was not required for this manuscript as all included intervention data has been previously published with ethical approval.

### Eligibility criteria

We included all randomised control trials that compared metformin with placebo in people without diabetes mellitus who were on clozapine. The study had to report on the people on clozapine separately from other antipsychotics. Outcomes of interest included the following: weight, BMI, waist circumference, fasting blood glucose, blood insulin levels, homeostatic model assessment (HOMA), triglycerides, high-density lipoprotein (HDL), low-density lipoprotein (LDL), and diastolic and systolic blood pressure. Published data in all languages were included and translated into English.

### Search Strategy

We searched Pubmed, Embase, the Cochrane Schizophrenia Group’s Trials Register, as well as the following Chinese databases: the Chinese Biomedical Literature Service System and China Knowledge Resource Integrated Database from inception to 13 November 2015. We also did hand searches of the references lists of included studies and other key publications. Studies were limited to humans. In the case of Pubmed, we used the following search terms: (randomized controlled trial OR controlled clinical trial OR randomized OR placebo OR clinical trials OR randomly OR trial) AND (clozapin* OR clozaril OR zaponex OR denzapin* or clopine) AND metformin AND ((((((clozapin*) OR clozaril) OR zaponex) OR denzapin*) or clopine) AND antipsychotic[MeSH Terms]).

### Study selection

Studies were included if they were randomised controlled trials. The study had to include participants on clozapine who did not have an existing diagnosis of diabetes mellitus. The study had to have a metformin intervention arm and placebo control arm. Any psychosocial health intervention, if provided, had to be available to both the metformin and placebo groups. Outcome data had to include at least one of weight, BMI, waist circumference or fasting glucose.

All studies identified in Pubmed, Embase, the Cochrane Schizophrenia Group’s Trials Register were screened at the title and abstract level by two authors (DS and DW). All studies identified in Chinese databases were screened at the title and abstract level by two authors (DS and JL). Studies that met the inclusion criteria on title and abstract review, or that could not be excluded on the basis of information provided in the abstract were reviewed at full text level. Snowball searches of key papers and the included studies’ reference lists were conducted. Narrative and systematic reviews, posters, conference abstracts, case reports, letters to editors and other articles that did not meet the inclusion criteria were cross-referenced for additional potential sources of RCTs. Attempts were made to contact corresponding authors of included studies in cases where information was missing, particularly when a study included participants on clozapine, but did not specifically report data on this group separately from other antipsychotics.

### Data collection process

Data extraction from the English language studies was conducted by two independent researchers (DS and DW), while data from the Chinese database studies was extracted by JL. All discrepancies during all stages of study selection, data extraction, and quality assessment were resolved by re-checking source papers. Data analysis was conducted by two authors (DS and SK).

### Data Items

We extracted data on the following outcomes of interest: weight, BMI, waist circumference, fasting blood glucose, blood insulin levels, homeostatic model assessment (HOMA), triglycerides, high-density lipoprotein (HDL), low-density lipoprotein (LDL), and diastolic and systolic blood pressure. Data was also extracted on trial duration, country, setting, diagnostic tool, whether participants were newly commenced on clozapine, mean age for the intervention and control groups, number of participants on metformin and placebo, gender of participants and mean dose of metformin.

### Outcomes

Where multiple outcome time points were reported in the same study, the data for the last outcome time point was used. Studies reported either mean endpoint outcome and standard deviation (SD) or mean change in outcome and SD.

The primary outcome was change in weight over time in kilograms (kg). Secondary outcomes were BMI, waist circumference in centimeters (cm), fasting blood glucose in milligrams per deciliter (mg/dL), triglycerides in milliMolar per Litre (mmol/L), high-density lipoprotein (HDL) (mmol/L), diastolic and systolic blood pressure in millimeters of mercury (mmHg), low-density lipoprotein (LDL) (mmol/L) blood insulin levels in milliUnits per Litre (mU/L), and homeostatic model assessment (HOMA).

### Study quality

Quality of included studies was assessed using the following criteria adapted from Cochrane Collaboration guidelines: 1) adequate generation of allocation sequence; 2) blinding of allocation to conditions to participant and/or assessor; 3) adequate random sequence generation; 4) pre-specified primary outcome measures; 5) appropriate reporting on missing data; 6) use of intention to treat analysis, and; 7) other sources of potential bias including pharmaceutical company funding.

### Statistical Analyses

Review Manager version 5.3 for Mac was used for the meta-analyses. Where possible, intention-to-treat analyses (ITT) were used. Difference in means was the principal summary measure for all analyses except dropouts, for which risk ratio was used.

We conducted sensitivity analyses on study quality, database source, whether or not the study included a psychosocial weight loss program in conjunction with both metformin and placebo, and whether the study endpoint was less than three months.

We assessed heterogeneity using the I^2^ statistic, a measure that does not depend on the number of studies in the meta-analysis and hence has greater power to detect heterogeneity when the number of studies is small. I^2^ provides an estimate of the percentage of variability due to heterogeneity rather than chance alone. An estimate of 50% or greater indicates possible heterogeneity, and scores of 75–100% indicate considerable heterogeneity. The I^2^ estimate is calculated using the chi-squared statistic (Q) and its degrees of freedom [19].

The random effects model was used for all the analyses as we could not definitely exclude between-study variation even in the absence of statistical heterogeneity given the range of medications under review. For any meta-analysis where there were more than ten studies, we tested for publication bias using funnel plot asymmetry where low P-values suggest publication bias [19].

## Results

### Study selection

**Fig 1:**
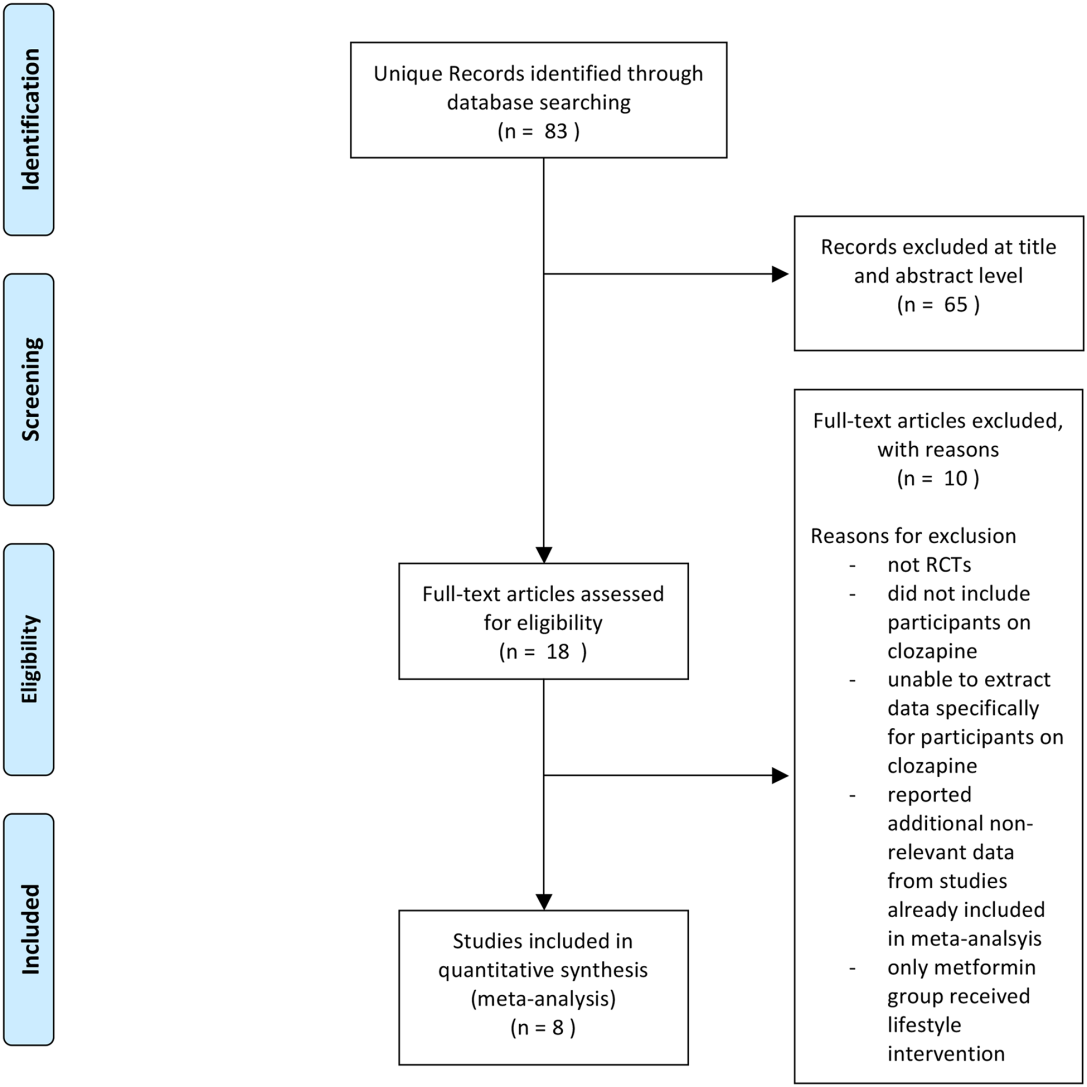
PRISMA Flow Diagram.

Eighty-three unique articles were identified in the initial search of the electronic databases (of which 24 were in Chinese databases). Of these, 65 were excluded at title and abstract level. Eight were further excluded after review of the full text, with reasons given in Fig 1. Although ten articles included people on clozapine, only eight provided separate data for clozapine and so could be included in the review [20–27]. No additional articles were identified in the hand searches.

### Study characteristics

Characteristics of the included studies are presented in Table 1. Of note, none of the studies looked at clozapine-naïve participants, and all but two were conducted exclusively in a hospital setting. Six out of the eight studies were conducted in China or Taiwan. One study, identified from the Chinese databases had a psychosocial weight loss intervention for both the metformin and placebo groups [27]. Two studies, identified in non-Chinese databases, had a psychosocial weight loss intervention as one component of the study, and included participants on clozapine and other antipsychotics [24,25]. The author of these studies kindly provided data for just the participants on clozapine, and only for the metformin and placebo arms that did not involve a psychosocial weight loss intervention.

**Table 1:** Included Studies.

Paper^1^	Duration^2^	Country	Setting^3^	Diagnostic tool^4^	Diagnoses	Participants already on clozapine (Y/N)	Presence of Psychosocial weight loss intervention	Mean Age (SD) for Metformin / Placebo^5^	Mean baseline weight (SD) for Metformin / Placebo^5^	Number of Participants Metformin / Placebo	Number Completed Metformin / Placebo	Male %	Mean Daily Metformin dose	Allocation^6^	Blinding^7^	Randomisation^8^	Primary Outcome^9^	Reporting^10^	ITT^11^	Other Bias^12^
Carizzo et al 2009^13^	14 w	Venezuela	C	DSM-IV	Schizophrenia Bipolar I	Y	No	39.6 (9.7) / 38.3 (8.7)	82.2 (20.9) / 77.1 (15.8)	31 / 30	24 / 30	NS	1000 mg	Yes	Double	Yes	Yes	Yes	No	No
Chen et al 2013^13^	24 w	Taiwan	H+C	DSM-IV	Schizophrenia Schizoaffective	Y	No	41.8 (7.2) / 41.4 (10.2)	69.1 (13.7) / 67.2 (9.6)	28 / 27	28 / 27	51%	1500 mg	Yes	Double	Yes	Yes	Yes	Yes	No
Hebrani et al 2015^13^	20 w	Iran	H	DSM-IV-TR	Schizophrenia	Y	No	47.2 (10.4) / 45.8 (10.2)	78.44 (13.4) / 70.50 (17.02)	30 / 30	19 / 18	46%	1000 mg	NS	Double	Yes	Yes	Yes	No	No
Wu et al 2008^13^	12 w	China	H	DSM-IV	Schizophrenia	Y	No^14^	26.8 / 25.8^15^	64.7 / 64.6^15^	11 / 10^16^ (Clozapine subgroup)	11 / 10^16^ (Clozapine subgroup)	NS	750 mg	Yes	Double	Yes	Yes	Yes	Yes	No
Wu et al 2012^13^	6 m	China	H	DSM-IV	Schizophrenia	Y	No	25.7 (4.8) / 27.1 (4.2) ^15^	56.6 (5.2) / 56.8 (5.6) ^15^	2 / 1^16^ Clozapine subgroup)	2 / 1^16^ (Clozapine subgroup)	0%	1000 mg	Yes	Double	Yes	Yes	Yes	Yes	No
Liu 2012^17^	12 w	China	H	CCMD-3	Schizophrenia	NS	No	31.5 (8.6) / 29.8 (6.5)	60.5 (8.3) / 62.3 (9.6)	50 / 50	50 / 50	61%	250–500^18^ mg-d	No	No	Yes	Yes	Yes	No	FNS
Wang 2009^17^	6 m	China	H	CCMD-3	Schizophrenia	Y	For both Metformin and Placebo	38 (16) / 37 (15)	NS	71 / 71	69 / 67	63%	1500 mg	No	No	Yes	Yes	Yes	No	FNS
Wu et al 2014^13^	12 w	China	H	CCMD-3	Schizophrenia	Y	No	27.22 (6.40) / 25.80 (6.23)	61.8 (7.3) / 61.9 (7.4)	36 / 36	36 / 36	59%	1000mg	No	No	Yes	Yes	Yes	No	FNS

NS = not stated

^1^. Lead author and year of publication

^2^. w = weeks, m = months

^3^. H = Hospital, C = Community

^4^. DSM = Diagnostic and Statistical Manual CCMD-3 = Chinese classification of mental disorder

^5^. SD = Standard Deviation,

^6^. Adequate allocation concealment

^7^. Single is to assessor only

^8^. Adequate Random Sequence Generation

^9^. Primary Outcome Measures were pre-specified and reported

^10^. Completeness of outcome reporting

^11^. ITT = Intention to Treat analysis

^12^. Were other potential sources of bias present. FNS = funding source not specified

^13^. Identified through search of western databases

^14^. Only data for the participants in the metformin and placebo without psychosocial health intervention groups were included.

^15^. Mean and SD for participants on all anti-psychotics, not just participants on clozapine

^16^. Only participants on clozapine included in meta-analysis

^17^. Identified through search of Chinese databases

^18^. Range, no mean reported

Study quality was fair for the five studies identified through non-Chinese databases, but was poor for the three studies identified in Chinese databases. Four non-Chinese database and no Chinese database papers reported adequate allocation concealment, and five non-Chinese database and no Chinese database papers were double blinded. Adequate random sequence generation was reported in all papers. Primary outcome measures were reported in all papers. Missing data were adequately described in all papers. Only three non-Chinese database and no Chinese database papers reported intention to treat data. No Chinese database papers provided information of the funding source, while no non-Chinese database paper reported financial support from a pharmaceutical company.

Eight studies were included in the meta-analysis, providing data for 239 people on metformin and 239 people on placebo. Studies were published between 2008 and 2015. Studies reported time points from six weeks to six months follow-up.

A meta-analysis of baseline weight and age of participants showed no statistically significant difference between metformin and placebo groups.

### Change in Weight

**Fig 2:**
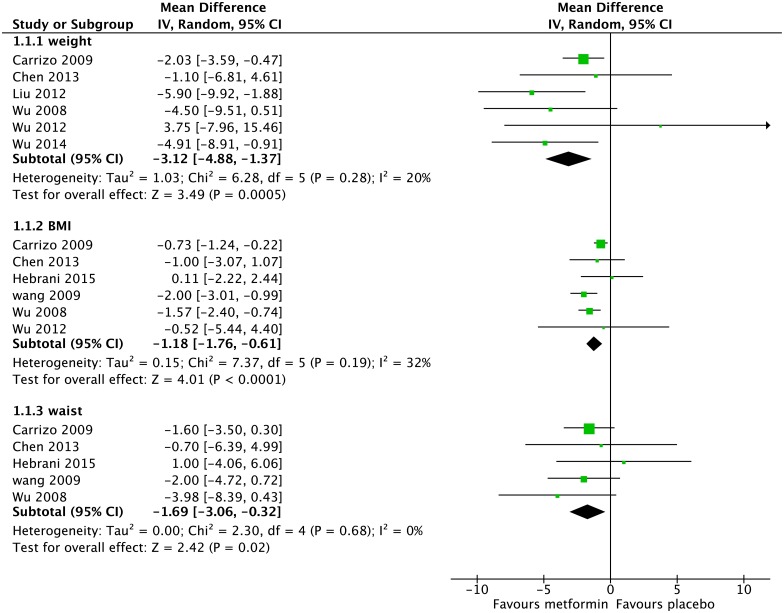
Forest Plot of Weight, BMI and Waist Circumference*. *weight = body weight in kg; BMI = Body Mass Index in kg/m2; Waist = waist circumference in cm.

Seven studies had usable data for change in weight with 151 people on metformin and 154 people on placebo (Fig 2). There was a significantly greater weight loss among the metformin group (-3.12kg, 95%CI -4.88kg to -1.37kg, Z = 3.49, p = 0.0005).

For Body Mass Index (BMI), six studies had usable data with 153 people on metformin and 153 people on placebo (Fig 2). BMI was significantly lower for the metformin group (-1.18kg/m^2^, 95%CI -1.76kg/m^2^ to -0.61kg/m^2^, Z = 4.01, p<0.0001).

### Change in Metabolic Syndrome components

#### Waist circumference

Five studies provided usable data on waist circumference with 151 people on metformin and 152 people on placebo (Fig 2). In the metformin group, waist circumference was 1.69cm less than in the placebo group (95%CI -3.06cm to -0.32cm, Z = 2.42, p = 0.02).

#### Glucose

**Fig 3:**
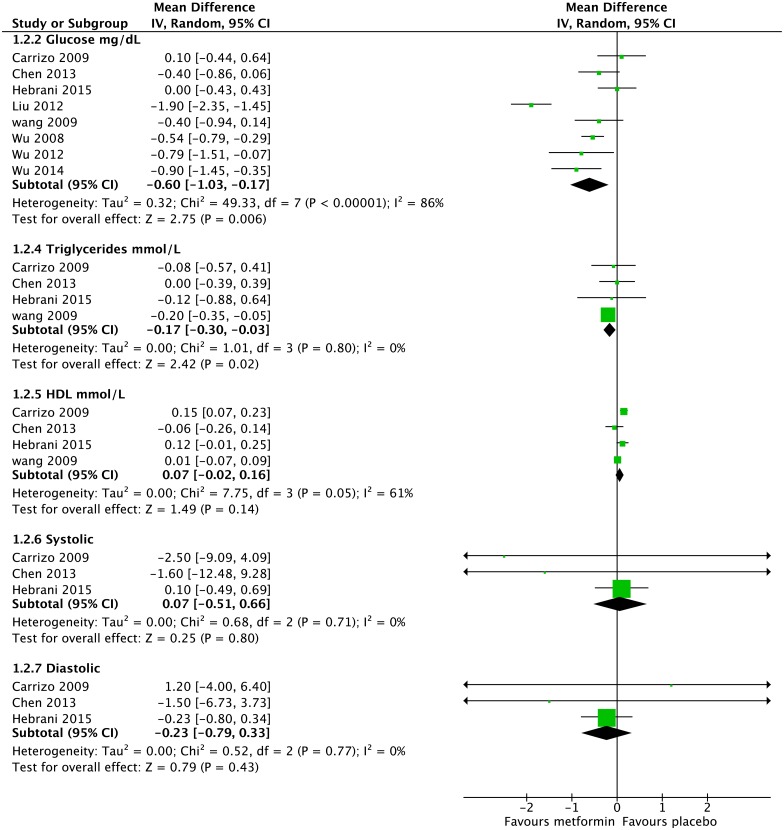
Forest Plot of Metabolic Syndrome Components (excluding Waist Circumference). HDL = High Density Lipoprotein; Systolic = Systolic Blood Pressure (in mmHg); Diastolic = Diastolic Blood Pressure (in mmHg).

For fasting blood glucose, eight studies had usable data for 239 people on metformin and 239 people on placebo (Fig 3). The metformin group had 0.60mg/dL lower fasting blood glucose than the placebo group (95% CI -1.03mg/dL to -0.17mg/dL, Z = 2.75, p = 0.006).

#### Triglycerides

Four studies, all with endpoints greater than three months, provided usable data for triglycerides, with 140 people on metformin and 142 on placebo (Fig 3). People on metformin had -0.17mmol/L lower triglyceride levels that people on placebo (-0.30mmol/L to -0.03mmol/L, Z = 2.42, p = 0.02).

#### HDL

For HDL, four studies, all with endpoints greater than three months, provided usable data with 140 people on metformin and 142 on placebo (Fig 3). There was no significant difference between the metformin and placebo groups.

#### Blood pressure

Three studies, all with endpoints greater than three months, provided usable data for blood pressure, with 71 people on metformin and 75 people on placebo (Fig 3). There was no significant difference between the groups for either systolic or diastolic blood pressure.

### Other Results

#### LDL

**Fig 4:**
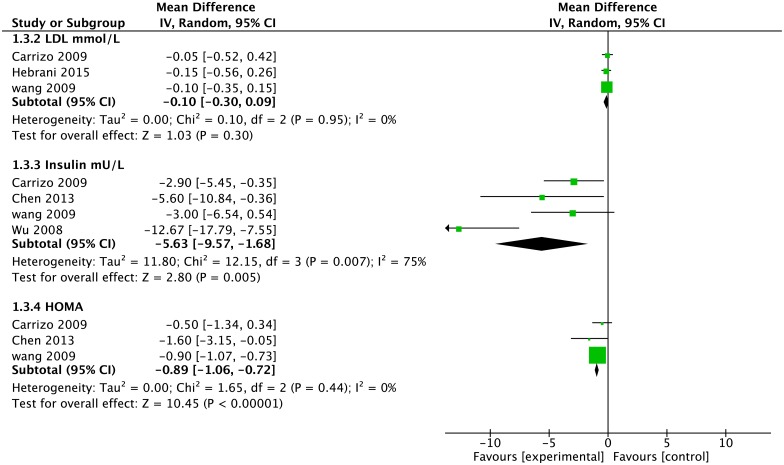
Forest Plot of LDL, Insulin and HOMA*. * LDL = Low Density Lipoprotein; HOMA = Homeostatic Model Assessment.

Data on LDL was available from three studies, all with endpoints greater than three months, with 112 people on metformin and 115 people on placebo (Fig 4). There was no statistically significant difference between the metformin and placebo groups.

#### Insulin

Four studies provided usable data on insulin levels for 132 people on metformin and 134 people on placebo (Fig 4). Insulin levels were 5.63mU/L lower for the metformin group (95% CI -9.57 to -1.68, Z = 2.80, p = 0.005).

#### HOMA

Data on HOMA was available from three studies, all with endpoints greater than three months, with 121 people on metformin and 124 people on placebo (Fig 4). HOMA was 0.89 lower for the metformin group (95% CI -1.06 to -0.72, Z = 10.45 p<0.0001).

### Dropouts

There was no statistically significant difference between the metformin and placebo groups in terms of dropouts, however only three studies reported any dropouts in either the metformin or placebo groups.

### Sensitivity analyses

Restricting the analyses to better quality studies, all of which were sourced from the non-Chinese databases, did not alter the results for weight, BMI, waist circumference, glucose, LDL or Insulin. There was no difference for weight, BMI, waist circumference, blood pressure, LDL or insulin when excluding studies of less than three months, or removing the study where clozapine or placebo were added to a psychosocial weight loss intervention. These results are summarised in S1 Table. For example, weight loss was 2.09kg, (95%CI -3.51kg to -0.66kg, Z = 2.86, p = 0.004) when the two poor quality studies were excluded, and 1.87kg, (95%CI -3.36kg to -0.38kg, Z = 2.46, p = 0.01) when three studies reporting data less than three months were excluded. There were no studies including a lifestyle intervention that provided usable data on weight.

However, sensitivity analyses did alter the results for other outcomes. For example, removing studies of less than three months meant the results for glucose were no longer statistically significant (-0.25mg/dL, 95%CI -0.53 to 0.03, Z = 1.74, p = 0.08). Similarly restricting the analyses to better quality studies altered the results for triglycerides (-0.04, 95% -0.33 to 0.24, Z = 0.30, p = 0.76 and HOMA (-0.85, 95%CI -1.85 to 0.15, Z = 1.66, p = 0.10).

By contrast, removing one poor quality study that included a psychosocial weight loss intervention changed the non-significant results for HDL with people on metformin having significantly higher HDL levels than placebo (0.10mmol/L, 95%CI 0.00mmol/L to 0.20mmol/L, Z = 1.93, p = 0.05)

### Publication Bias

There were insufficient studies to test for publication bias.

## Discussion

This study is the first systematic review and meta-analysis to specifically examine the impact of metformin on weight, BMI and metabolic syndrome components for people on clozapine. Previous systematic reviews have considered antipsychotic agents in general [11,28,29]. One did include a sub-analysis of people on clozapine for a restricted range of variables [11]. We were able to include three additional studies and conduct analyses on all components of metabolic syndrome. A particular strength was the use of Chinese databases to search for papers that did not appear in the Cochrane Schizophrenia Group’s trial register, PubMed or Embase. There is increasing discussion in the epidemiological literature regarding searches of Chinese databases [30,31]. By accessing Chinese databases we were able to identify three additional studies and thus able to include eight studies with 478 participants in the meta-analysis. Although the studies accessed from Chinese databases had a higher risk of bias than those identified through the other databases, sensitivity analyses of excluding them did not alter the significance of most variables.

We found that metformin was superior to placebo in terms of weight loss and BMI, and that this weight loss was clinically meaningful. St Jeor et al (1999) defined a clinically meaningful weight change as being greater than 2.3kg,based on findings from a US observational study of adults [32]. Metformin also significantly improved three of the five components of metabolic syndrome, namely, waist circumference, fasting glucose and triglycerides.

Study duration did not appear to have a large impact on the results, with the exclusion of studies with endpoints less than three months only altering the significance for fasting glucose. This is in keeping a previous large study of people with type 2 diabetes not on clozapine, with fasting glucose and HbA1c remaining stable after 5 weeks to the study endpoint of 29 weeks [33].

There were several limitations of this study. All but two of the studies drew participants exclusively from a hospital setting. It is possible that the controlled environment of a hospital may differ in terms of diet and access to exercise when compared to a community setting. In addition, participants in all but two of the studies were from China and Taiwan; this may affect the generalisability of our findings given that the criteria for metabolic syndrome, notably waist circumference, differ between Caucasian and Asian populations [8]. In this regard, an earlier small meta-analysis of metformin for weight loss among people on antipsychotics noted a larger magnitude of weight loss among Asian compared to Hispanic populations [28]. There were insufficient data to comment on rates of adverse drug reactions between the metformin and placebo groups. However we did assess for drop-outs and, importantly, there was no statistically significant difference in discontinuation rates between the groups. Finally, many of our results showed heterogeneity. We conducted sensitivity analyses and have used a random effects model throughout to incorporate heterogeneity into our analysis, however these results should still be treated with caution. Only one of the included studies reported on whether study participants met threshold criteria for the metabolic syndrome components at the end of the study period [21], and as such, we were unable to conduct a meaningful meta-analysis. We are unable to comment on whether metformin has an impact on resolution of individual abnormal metabolic syndrome components or on total criteria for metabolic syndrome.

Although these results suggest modest reversal of clozapine associated weight gain, primary prevention of weight gain would be more valuable. Further research into the concomitant commencement of clozapine and metformin for the prevention of weight gain is needed. Additionally, examination of other hypoglycaemic agents, such as GLP-1 agonists (e.g. exenatide) [17,34] for weight loss for people on clozapine may provide additional weight loss options.

Given the promising results for metformin for weight loss for people on clozapine, pathways to metformin commencement need to be examined. Traditionally, psychiatrists have prescribed non-psychiatric medications for management of antipsychotic ADRs, such as the use of benztropine for antipsychotic induced extrapyramidal side effects. With appropriate training, psychiatrists should be competent to prescribe metformin for people on clozapine with metabolic syndrome but without diabetes. For psychiatrists who are not yet comfortable to directly prescribe metformin, they should be building strong collaborative partnerships with primary care, and encouraging their primary care colleagues to prescribe metformin.

### Conclusions

The clinically relevant weight loss of over 3kg associated with metformin use suggests that metformin could be an appropriate adjuvant for people on clozapine who are overweight and obese. It may be worth adding metformin, as tolerated, to the treatment protocols for people with obesity on clozapine.

The impact on metabolic syndrome components is also promising. Further research is needed to examine the effect of concomitant commencement of clozapine and metformin versus clozapine and placebo to prevent weight gain and the development of type 2 diabetes in clozapine naïve people with treatment refractory schizophrenia.

## Supporting Information

S1 Table Sensitivity Analysis. * All high quality studies were sourced from the non-Chinese databases; HDL = High Density Lipoprotein; LDL = Low Density Lipoprotein.
(DOCX)

S2 Table PRISMA Checklist. (DOC)
