# Supplementary material for: Metformin for Clozapine Associated Obesity: A Systematic Review and Meta-Analysis
Source: PLoS One. 2016 Jun 15;11(6):e0156208. doi: 10.1371/journal.pone.0156208 (PMC4909277; doi:10.1371/journal.pone.0156208)
Supplement: S1 Table — * All high quality studies were sourced from the non-Chinese databases; HDL = High Density Lipoprotein; LDL = Low Density Lipoprotein. (DOCX) [file pone.0156208.s001.docx]

**S1 Table. Sensitivity Analyses**

| Analysis | Number of studies | Difference | 95% CI | Z value | p value |
| --- | --- | --- | --- | --- | --- |
| Restricted to High Quality Studies* |  |  |  |  |  |
| Weight | 4 | -2.09Kg | -3.51 to -0.66 | 2.86 | 0.004 |
| BMI | 5 | -0.93kg/m^2^ | -1.34 to -0.51 | 4.33 | <0.0001 |
| Waist Circumference | 4 | -1.58cm | -3.17 to 0.00 | 1.96 | 0.05 |
| Glucose | 5 | -0.32mg/dl | -0.61 to -0.02 | 2.10 | 0.04 |
| Triglycerides | 4 | -0.04mmol/L | -0.33 to 0.24 | 0.30 | 0.76 |
| HDL | 3 | 0.10mmol/L | 0.00 to 0.20 | 1.96 | 0.05 |
| LDL | 2 | -0.11mmol/L | -0.41 to 0.20 | 0.68 | 0.50 |
| Insulin | 3 | -6.77mU/L | -12.59 to -0.96 | 2.28 | 0.02 |
| HOMA | 2 | -0.85 | -1.85 to 0.15 | 1.66 | 0.10 |
| Restricted to outcome reported at greater than 3 months |  |  |  |  |  |
| Weight | 3 | -1.87 | -3.36 to -0.38 | 2.46 | 0.01 |
| BMI | 5 | -1.05kg/m^2^ | -1.77 to -0.34 | 2.89 | 0.004 |
| Waist Circumference | 4 | -1.44cm | -2.88 to 0.00 | 1.96 | 0.05 |
| Glucose | 5 | -0.25 | -0.53 to 0.03 | 1.74 | 0.08 |
| Insulin | 3 | -3.29 | -5.22 to -1.37 | 3.36 | 0.0008 |
| HOMA |  |  |  |  |  |
| Exclusion of psychosocial weight loss intervention |  |  |  |  |  |
| BMI | 5 | -0.93kg/m^2^ | -1.34 to -0.51 | 4.33 | <0.0001 |
| Waist Circumference | 4 | -1.58cm | -3.17 to 0.00 | 1.96 | 0.05 |
| Glucose | 7 | -0.63mg/dl | -0.11 to -0.15 | 2.56 | 0.01 |
| Triglycerides | 4 | -0.04mmol/L | -0.33 to 0.24 | 0.30 | 0.76 |
| HDL | 3 | 0.10mmol/l | 0.00 to 0.20 | 1.96 | 0.05 |
| LDL | 2 | -0.11 | -0.41 to 0.20 | 0.68 | 0.50 |
| Insulin | 3 | -6.77mU/L | -12.59 to -0.96 | 2.28 | 0.02 |
| HOMA | 2 | -0.85 | -1.85 to 0.15 | 1.66 | 0.10 |

* All high quality studies were sourced from the non-Chinese databases

HDL = High Density Lipoprotein

LDL = Low Density Lipoprotein
